# Cataract, Visual Impairment and Long-Term Mortality in a Rural Cohort in India: The Andhra Pradesh Eye Disease Study

**DOI:** 10.1371/journal.pone.0078002

**Published:** 2013-10-25

**Authors:** Rohit C. Khanna, Gudlavalleti V. S. Murthy, Pyda Giridhar, Sannapaneni Krishnaiah, Hira B. Pant, Ghanshyam Palamaner Subash Shantha, Subhabrata Chakrabarti, Clare Gilbert, Gullapalli N. Rao

**Affiliations:** 1 Allen Foster Research Centre for Community Eye Health, International Centre for Advancement of Rural Eye care, L V Prasad Eye Institute, Hyderabad, India; 2 Brien Holden Eye Research Centre, L.V. Prasad Eye Institute, Hyderabad, India; 3 International Centre for Eye Health, Department of Clinical Research, London School of Hygiene and Tropical Medicine, London, United Kingdom; 4 Indian Institute of Public Health, Hyderabad, India; 5 Johns Hopkins Bloomberg School of Public Health, Baltimore, Maryland, United States of America; 6 Department of Internal Medicine, Wright Center for Graduate Medical Education, Scranton, Pennsylvania, United States of America; Zhongshan Ophthalmic Center, China

## Abstract

**Background:**

A large-scale prevalence survey of blindness and visual impairment (The Andhra Pradesh Eye Diseases Study [APEDS1]) was conducted between 1996-2000 on 10,293 individuals of all ages in three rural and one urban clusters in Andhra Pradesh, Southern India. More than a decade later (June 2009-March 2010), APEDS1 participants in rural clusters were traced (termed APEDS2) to determine ocular risk factors for mortality in this longitudinal cohort.

**Methods and Findings:**

Mortality hazard ratio (HR) analysis was performed for those aged >30 years at APEDS1, using Cox proportional hazard regression models to identify associations between ocular exposures and risk of mortality. Blindness and visual impairment (VI) were defined using Indian definitions. 799/4,188 (19.1%) participants had died and 308 (7.3%) had migrated. Mortality was higher in males than females (p<0.001). In multivariable analysis, after adjusting for age, gender, diabetes, hypertension, body mass index, smoking and education status the mortality HR was 1.9 (95% CI: 1.5-2.5) for blindness; 1.4 (95% CI: 1.2-1.7) for VI; 1.8 (95% CI: 1.4-2.3) for pure nuclear cataract, 1.5 (95% CI: 1.1-2.1) for pure cortical cataract; 1.96 (95% CI: 1.6-2.4) for mixed cataract, 2.0 (95% CI: 1.4-2.9) for history of cataract surgery, and 1.58 (95% CI: 1.3-1.9) for any cataract. When all these factors were included in the model, the HRs were attenuated, being 1.5 (95% CI: 1.1-2.0) for blindness and 1.2 (95% CI: 0.9-1.5) for VI. For lens type, the HRs were as follows: pure nuclear cataract, 1.6 (95% CI: 1.3-2.1); pure cortical cataract, 1.5 (95% CI: 1.1-2.1); mixed cataract, 1.8 (95% CI: 1.4-2.2), and history of previous cataract surgery, 1.8 (95% CI: 1.3-2.6).

**Conclusions:**

All types of cataract, history of cataract surgery and VI had an increased risk of mortality that further suggests that these could be potential markers of ageing.

## Introduction

There has been inconsistency in the association between mortality and visual impairment (VI) [1-12] with some studies demonstrating a positive association [2-4,6-11], whereas others do not [1,5,12]. Similar findings have been reported for cataract [1-6,8,10-19], diabetic retinopathy (DR) [6,11,12,16] and age related macular degeneration (ARMD) [1-3,5,6,8,10-13,16]. These inconsistencies could be explained by differences in study design, age of the study participants, follow-up intervals, the definitions used and in the analytical approach to controlling for potential confounders. 

Different biological mechanisms have been proposed for the association between mortality and certain eye diseases [20-24]. Possible explanations are that eye diseases such as cataract are markers of biological aging and that common ocular conditions may share a common attribute with conditions associated with increased mortality [20,22]. Another possible reason is that the excess mortality is a consequence of VI, which increases the risk of falls and depression, particularly in the elderly [21].

As there is a paucity of data on the risk of mortality among those who are blind and visually impaired from India, we explored this association through a follow-up study of participants in the Andhra Pradesh Eye Disease Study (APEDS) [25].

## Material and Methods

This study was approved by the Institutional Review Board of L V Prasad Eye Institute and was conducted adhering to the Tenets of the Declaration of Helsinki. This study was part of a tracing exercise of a longitudinal cohort (that was studied more than a decade ago), in order to identify participants who were available for a reexamination. The causes of death were noted for participants who had died during this period. Since the majority of the participants were not literate enough to either read or sign the consent form, a verbal consent was obtained in the presence of the village head after explaining the purpose of this exercise to them. This process was documented in our study protocol and was further approved by the Ethics Committee of the L.V. Prasad Eye Institute. For brevity, the original APEDS survey will be called APEDS1 and the recent study described here will be APEDS2, which was limited to individuals aged 30 years or above at the time of APEDS1. 

### Methods used in APEDS1

The initial Andhra Pradesh Eye Disease Study (APEDS1) has been described elsewhere [25,26]. Briefly, the study was conducted in the South Indian state of Andhra Pradesh (AP) between 1996 and 2000 in one urban and three rural areas representative of the population of the State. The principle aim of APEDS was a) to estimate the prevalence of eye diseases, blindness and VI, b) to determine risk factors associated with the major eye diseases, c) to assess the effect of blindness and VI on quality of life (QoL) and d) to investigate barriers to the uptake of services. The study sample consisted individuals of all ages and was selected using stratified random sampling with probability proportionate to size procedures so that each social group (caste) was adequately represented in each area. The sample size (n = 10,293) was calculated to give a precise estimate of the prevalence of blindness of 0.5% for eye disease of least prevalence in those aged ≤30 years (ocular trauma) and >30 years (ARMD). Hence, a sample size of 5000 in each of these two age groups (≤ 30 years and >30 years) was chosen such that the estimation of these diseases in their respective age group would have similar precision. Participants were recruited from four areas, one urban area (Hyderabad, n=2,522) and three rural areas, namely Tanuku (West Godavari district, n=2,503), Mudhole (Adilabad district, n=2690), and Thoodukurthy (Mahabubnagar district, n=2578). 

Interviews, standard eye examinations, and anthropometric measurements such as blood pressure, weight and height were undertaken at examination sites set up for the study. Participants aged more than 15 years were administered a structured questionnaire by trained interviewers to collect information on risk factors for eye diseases (e.g. severe diarrhoea), presence of systemic diseases (e.g. hypertension, diabetes, leprosy), behaviours (e.g. current or previous smoking, alcohol consumption, or chewing tobacco) and regular use of medication (steroids, multivitamins etc). Clinical examinations were performed by a team comprising four ophthalmologists and four optometrists who had undergone a detailed training. 

Standard examination included distance and near visual acuity (VA), both presenting (PVA) and best corrected (BCVA) after refraction, measured for each eye separately using logMAR (logarithm of minimum angle of resolution) charts. All subsequently underwent a full ophthalmic examination including slit lamp biomicroscopy, dilated fundus examination and lens grading using the Lens Opacities Classification System (LOCS-III) and the Wilmer classification [27,28]. Participants who were unable to visit the clinic due to physical ailment or other reasons were examined at home using similar methods. However, gonioscopy, examination with 78 diopter lens, automated visual fields, and photography could not be accomplished in these participants.

Blindness was defined using Indian definitions as PVA less than 6/60 or central visual field less than 20° in the better eye [29]. VI was defined as PVA less than 6/18-6/60 or equivalent visual field loss [30]. The lens was examined after fully dilating the pupils. Nuclear opacity was graded clinically using the Lens Opacity and Classification System III (LOCS III) [27] and cortical and posterior subcapsular cataract were graded using the Wilmer classification [28]. Because different types of cataract frequently co-exist, for analysis we considered pure nuclear, pure cortical, pure posterior subcapsular cataract (PSC) and mixed type of cataract. Those with total cataract were grouped under mixed cataract category and those having undergone cataract surgery (unilateral / bilateral) were treated as separate group. We defined the presence of pure nuclear cataract (NC) as at least one eye showing nuclear opalescence of grade 3.0 or higher on LOCSIII [31]. Pure cortical cataract (CC) was considered present if at least one eye had a Wilmer grade of 2 or higher. Pure PSC was considered present if at least one eye had a Wilmer grade of 1 or higher [31]. Pure nuclear, cortical or PSC subgroup had isolated cataract without the presence of other subtypes. Mixed cataract was defined as at least one eye showing combination of any two type of opacity and any cataract was defined as having any of the above mentioned opacity, including history of cataract surgery [31]. For analysis, the opacity in the most affected eye was used. ARMD was defined as based on International Classification and Grading System [32] and DR was defined based on the modification of the standard classification system [33]. 

Hypertension was defined as present if the participant gave a history of high blood pressure diagnosed by a physician and / or current treatment with antihypertensive medications and / or a had a blood pressure reading of ≥140/90 mm Hg. Diabetes was defined as present if a participant gave a history of diabetes and / or was taking diabetic medication and / or diabetic retinopathy was detected on clinical examination. The duration of diabetes since diagnosis was also documented. Body mass index (BMI) was calculated from the measured height and weight according to the formula weight (in kilograms) divided height (in meters) squared. World Health Organization categories were used i.e. underweight (BMI <18.5), normal (18.5 ≤ BMI < 25), over weight (25 ≤ BMI < 30), and obese (BMI ≥ 30) [34,35]. For smoking, participants were categorized as never smoker, former smoker and current smoker. Current and former smokers were those who had smoked for a minimum of 1 year. Participants who had never smoked, or had smoked for less than 1 year were considered “never smokers”. Details of smoking used in analysis have been published elsewhere [31].

The cause of visual loss (VI or blindness) in each eye was determined and documented by an ophthalmologist in consultation with the principal investigator. For instance, if cataract and a posterior-segment lesion of the optic nerve or retina coexisted and removal of cataract was unlikely to restore vision, the cause of blindness was attributed to the posterior-segment lesion. On the other hand, if dense cataract was present that prevented a clear view of the posterior segment and with no other signs suggestive of any other cause of visual loss, the cause of blindness was considered to be cataract. If index myopia was present due to cataract, and even if the vision improved with refraction, the cause of blindness was attributed to cataract as it was the major underlying cause. If the causes differed between eyes, the cause most amenable to treatment was selected as the cause of blindness for the person.

### Methods used in APEDS2

Extensive changes have taken place in urban and semi-urban Hyderabad over the last decade, and the original urban area could not be delineated. Hence, the tracing exercise, undertaken from 2009-2010 was limited to the rural clusters. The purpose of tracing the original participants was as follows:

1. To assess the mortality rate amongst those who were aged 30 years and above at APEDS1 and
2. To determine factors at APEDS1 that predicted subsequent mortality e.g. lens status, VI, ARMD, after adjusting for confounders

At APEDS1, 70 out of 94 clusters were included in the rural sample and 7,771 / 8,832 (88%) of those enumerated were clinically examined between 1996 and 2000. Of these, 4,188 (53.9%) were adults aged 30 years and above.

Names and addresses of APEDS1 participants were extracted from the existing database. The field investigators visited each cluster to gather further information on the APEDS I participants. Prior to field work, a pilot study was undertaken during which the instruments were standardized. Following this training phase, all surviving participants were interviewed in detail by trained field investigators using standard methods. In households where the original participant(s) had either died or migrated, a structured questionnaire was administered by trained interviewers to the present head of household to collect information on the cause and/or the reason of death and the year of death and migration, as applicable. In situations where the entire household had migrated, questions were administered to neighbors. As formal death certificates were not available, the cause of death was based on verbal autopsy using WHO recommended methods[36]. 

### Data Analysis

Data analysis was performed using STATA 11 [37]. For comparison of two continuous variables, student’s t test was used, while for multiple groups, one-way analysis of variance (ANOVA) was used. For categorical data, Fisher’s exact test was used and for analysis of risk factors for mortality and VI, cataract and ARMD, Cox-proportional hazard model was used [38]. Test of significance for survival curve was assessed using the log-rank test. Multi-colinearity between variables was assessed using variance inflation factors, and proportionality of the model was tested based on Schoenfeld residuals. Interaction effects were tested between gender and ocular parameters (VI, cataract and ARMD), diabetes and different morphologies of cataract (pure cortical, pure nuclear and mixed) and VI and cataract (pure nuclear cataract, pure cortical cataract, mixed cataract and history of cataract surgery). 

## Results

**Figure 1:**
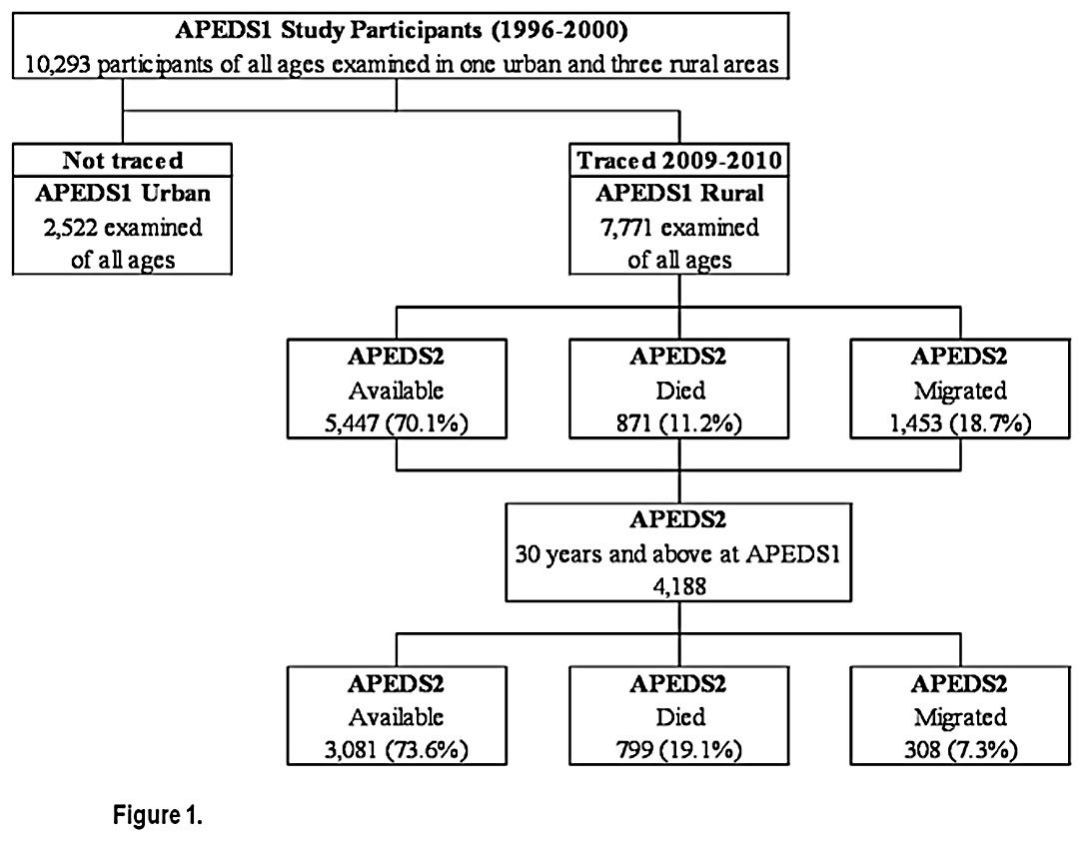
Availability status of participants at the time of APEDS2.

The interval between APEDS1 and APEDS2 ranged from 10-12 years (mean 11 years; SD: 0.81 year). Data were available on all the 4,188 individuals aged 30 years and above examined in rural clusters during APEDS1: 799 (19.1%) had died by APEDS2; 308 (7.3%) had migrated and 3,081 (73.6%) were still living in the area (i.e. they were “available”) (Figure 1). There were 1,964/4188 (46.9%) males in this dataset.

Migration was higher (53.9%) in females than males, and was higher in one of the rural areas (Mahabubnagar, 11.3%). There was no significant difference in the mean age of those available in APEDS2 (mean 45 years; SD: 11.22 years) compared to those who had migrated (mean 44.39 years; SD: 12.48 years; *P*=0.43), but those who had died were significantly older (mean 60.23 years; SD: 12.1 years; *P*<0.001).

Mortality rates were higher in males among those who were illiterate, smokers, had hypertension and diabetes (Table 1). There was no association between mortality and alcohol consumption (*P*=0.19).

**Table 1:** Distribution of various demographic, lifestyle and systemic risk factors for mortality in APEDS2.

**Risk Factors**	**Alive**	**Died**	**Total**
	**N (%)**	**N (%)**	**N (%)**
**Age group (years)**			
30 - 39	1338 (39.5)	60 (7.5)	1398 (33.4)
40 - 49	939 (27.7)	90 (11.3)	1029 (24.6)
50 -59	632 (18.7)	155 (19.4)	787 (18.8)
60 - 69	397 (11.7)	319 (39.9)	716 (17.1)
≥ 70	83 (2.5)	175 (21.9)	258 (6.2)
**Gender**			
Male	1533 (45.2)	431 (53.9)	1964 (46.9)
Female	1856 (54.8)	368 (46.1)	2224 (53.1)
**Education**			
Illiterate	2130 (62.9)	551 (69)	2681 (64)
Class 1-5*	707 (20.9)	173 (21.7)	880 (21.2)
Class 6-10	439 (13)	57 (7.1)	496 (11.8)
Class 11 and above*	113 (3.3)	18 (2.3)	131 (3.1)
**Hypertension**			
Absent	2230 (65.8)	410 (51.3)	2640 (63)
Present	1159 (34.2)	389 (48.7)	1548 (37)
**Diabetes**			
Absent	3345 (98.8)	763 (95.5)	4108 (98.1)
Present	42 (1.2)	36 (4.5)	78 (1.9)
**Smoking status** ^$^			
Never smoker	2817 (83.2)	603 (75.5)	3420 (81.7)
Former smoker	280 (8.3)	48 (6)	328 (7.8)
Current smoker	291 (8.6)	148 (18.5)	439 (10.5)
**Body Mass Index (BMI)**			
Normal	1636 (49.7)	295 (40.7)	1931 (48.1)
Under weight	1369 (41.6)	360 (49.7)	1729 (43.1)
Over weight*	236 (7.2)	55 (7.6)	291 (7.3)
Obese*	49 (1.5)	15 (2.1)	214 (1.6)
Alcohol consumption*			
Never Drinker	2254 (66.5)	515 (64.5)	2769 (66.1)
Former Drinker	836 (24.7)	221 (27.7)	1057 (25.2)
Current Drinker	298 (8.8)	63 (7.9)	361 (8.6)

* Not significant association seen. Rest all association were significant at *P*<0.05 level

^$^ Cigarette, Chuta (Indigenous Cigar) and Beedies smoking

Univariate analysis for eye diseases detected during APEDS1 showed that subsequent mortality was higher for those with ARMD and in those with blindness or VI. Individuals either with pure nuclear cortical, posterior subcapsular and mixed cataract, or a history of cataract surgery (irrespective of post-operative visual acuity) or any cataract also had a higher risk of mortality compared to those with minimal or no lens opacities at APEDS1 (Table 2)

**Table 2:** Distribution of cataract morphology and other ocular risk factors for mortality.

**Risk Factors**	**Alive**	**Died**	**Total**
	**N (%)**	**N (%)**	**N (%)**
**Visual acuity (VA)**			
6/6-6/18	2665 (78.7)	326 (40.9)	2991 (71.5)
<6/18-6/60	620 (18.3)	367 (46)	987 (23.6)
<6/60	101 (3)	105 (13.2)	206 (4.9)
**Pure Nuclear Cataract**			
Yes	194 (5.7)	160 (20)	354 (8.5)
No	3195 (94.3)	639 (80)	3834 (91.5)
**Pure Cortical Cataract**			
Yes	106 (3.1)	43 (5.4)	149 (3.6)
No	3283 (96.9)	756 (94.6)	4039 (96.4)
**Pure Posterior subcapsular cataract**			
Yes	74 (2.2)	27 (3.4)	101 (2.4)
No	3315 (97.8)	772 (96.6)	4087 (97.6)
**Mixed cataract**			
Yes	205 (6.1)	201 (25.2)	406 (9.7)
No	3184 (94)	598 (74.8)	3782 (90.3)
**History of cataract surgery**			
Yes	48 (1.4)	49 (6.1)	97 (2.3)
No	3335 (98.6)	749 (93.9)	4084 (97.7)
**Any cataract**			
Yes	627 (18.5)	480 (60.2)	1104 (26.4)
No	2756 (81.5)	318 (39.8)	3077 (73.6)
**ARMD** ^§^			
Present	32 (0.9)	22 (2.7)	54 (1.3)
Absent	3356 (99.1)	777 (97.3)	4133 (98.7)

All association were significant at *P*<0.05 level; ^§^ARMD: Age Related Macular Degeneration;

To further evaluate associations between ocular and systemic diseases, demographic and lifestyle factors with mortality, data were analysed using the Cox proportion hazard model. 

Univariable association of systemic disease, demographics, lifestyle factors and ocular factors with mortality is shown in tables 3 and 4. 

**Table 3:** Univariable association of systemic disease, demographic and lifestyle factors with mortality.

**Risk Factors**		**Univariable analysis**	
**Risk Factors Categories**		**Hazard ratio**	**95% CI**
Age group (yrs)			
	30 - 39	1.00	
	40 - 49	2.08	1.49, 2.88
	50 - 59	4.94	3.66, 6.66
	60 - 69	13	9.84, 17.15
	≥ 70	24.82	18.46, 33.38
**Gender**			
	Male	1.00	
	Female	0.73	0.63, 0.84
**Education**			
	Illiterate	1.00	
	Class 1 - 5	0.97	0.82, 1.16
	Class 6 - 10	0.56	0.43, 0.74
	Class 11 and above	0.74	0.47, 1.19
**Diabetes**			
	No	1.00	
	Yes	3.08	2.21, 4.31
**Hypertension**			
	No	1.00	
	Yes	1.7	1.48, 1.95
**Body Mass Index**			
	Normal	1.00	
	Underweight	1.39	1.19, 1.62
	Overweight	1.26	0.95, 1.69
	Obese	1.51	0.88, 2.58
Smoking status*			
	Never smoker	1.00	
	Former smoker	1.80	1.37, 2.38
	Heavy smoker	1.42	1.18, 1.71

* Cigarettes, Chuta (indigenous Cigar) and Beedies

**Table 4:** Univariable association of ocular factors with subsequent mortality.

**Risk Factors**	**Risk Factors categories**	**Hazard ratio**	**95% CI**
**Visual acuity**			
	≥6/18	1.00	
	<6/18 - 6/60	3.88	3.34, 4.51
	<6/60	6.27	5.03, 7.82
**ARMD^§^**			
	No	1.00	
	Yes	2.47	1.61, 3.77
**Pure nuclear cataract**			
	No	1.00	
	Yes	3.17	2.67, 3.77
**Pure cortical cataract**			
	No	1.00	
	Yes	1.55	1.14, 2.11
Pure PSC* cataract			
	No	1.00	
	Yes	1.5	1.02, 2.2
**Mixed cataract**			
	No	1.00	
	Yes	3.9	3.33, 4.58
**History of cataract surgery**			
	No	1.00	
	Yes	3.46	2.59, 4.64
**Any type of cataract, including cataract surgery**			
	No	1.00	
	Yes	5.1	4.41, 5.86

^§^ ARMD: Age Related Macular Degeneration; * PSC: Posterior Subcapsular Cataract

In the multivariable analysis, four models were considered. Model 1 included all the VI categories; model 2 included lens morphology and those who had undergone cataract surgery; model 3 included any cataract with all the VI categories and ARMD and model 4 included all the ocular factors (grades of VI, lens morphology, those who had undergone cataract surgery and ARMD). All these models were adjusted for systemic diseases, demographic and lifestyle factors (age, gender, education level, diabetes, hypertension, BMI and smoking status; Table 5). The findings were similar in Models 1, 2 and 3 in the univariate analysis, but the hazard ratios (HRs) were attenuated after adjusting for confounders. In Model 2, PSC was no longer associated with an increased risk for mortality (HR=1.00, 95% Confidence Interval (CI): 0.62, 1.58) and in Model 4, VI was no longer associated with mortality (HR=1.19, 95% CI: 0.97, 1.45). After adjusting for systemic diseases, demographic and lifestyle factors (age, gender, education level, diabetes, hypertension, BMI and smoking status), ARMD (HR=1.44, 95% CI: 0.92, 2.26) was no longer associated with mortality (data not shown). These associations were consistent even when age was used as a continuous variable or using the forward and backward stepwise method (data not shown).

**Table 5:** Multivariable association of ocular factors with mortality in four different models.

**Risk Factors**	**Risk Factors categories**	**Model 1^$^^All VI categories^**	**Model 2^$^^Lens morphology^**	**Model 3^$^^VI, any cataract and ARMD^**	**Model 4^$^^VI, lens morphology and ARMD^**
		HR (95% CI)	HR (95% CI)	HR (95% CI)	HR (95% CI)
**Visual acuity**					
	≥6/18	1.00 (Ref)	----	1.00 (Ref)	1.00 (Ref)
	<6/18-6/60	1.42 (1.18, 1.69)*	----	1.23 (1.01-1.48)*	1.19 (0.9, 1.45)
	<6/60	1.89 (1.45, 2.47)*	----	1.57 (1.19-2.07)*	1.46 (1.1, 1.95)*
**Schoenfeld test ^#^**		0.15	----	----	----
**ARMD^§^**					
	No	----	----	1.00 (Ref)	1.00 (Ref)
	Yes	----	----	1.26 (0.8-1.98)	1.25 (0.79, 1.97)
**Pure nuclear cataract**					
	No	----	1.00 (Ref)	----	1.00 (Ref)
	Yes	----	1.8 (1.43, 2.27)*	----	1.61 (1.25, 2.07)*
**Pure cortical cataract**					
	No	----	1.00 (Ref)	----	1.00 (Ref)
	Yes	----	1.48 (1.05, 2.08)*	----	1.49 (1.06, 2.09)*
Pure PSC* cataract					
	No	----	1.00 (Ref)	----	1.00 (Ref)
	Yes	----	1.00 (0.62, 1.58)	----	0.97 (0.61, 1.54)
**Mixed cataract**					
	No	----	1.00 (Ref)	----	1.00 (Ref)
	Yes	----	1.96 (1.57, 2.44)*	----	1.75 (1.38, 2.23)*
**Previous cataract surgery**					
	No	----	1.00 (Ref)	----	1.00 (Ref)
	Yes	----	2.01 (1.41, 2.88)*	----	1.82 (1.26, 2.62)*
**Schoenfeld test^#^**		----	0.31	----	0.08
**Any type of cataract, including cataract surgery**					
	No	----	----	1.00 (Ref)	----
	Yes	----	----	1.58 (1.30-1.92)*	----
**Schoenfeld test^#^**		----	0.31	0.12	----

^$^ Adjusted for age, gender, education level, diabetes, hypertension, BMI and smoking status.

^§^ ARMD: Age Related Macular Degeneration; * PSC: Posterior Subcapsular Cataract ^#^ goodness-of fit test; Ref: Reference group; * statistically significant (p<0.05)

No interactions were observed between gender, ocular parameters (VI, cataract and ARMD) and mortality risk or between diabetes and different morphology of cataract (pure cortical, pure nuclear and mixed). Similar results were observed with VI and different lens morphology (pure nuclear cataract, pure cortical cataract, mixed cataract) and history of cataract surgery. 

Many relatives reported symptoms rather than diagnoses and so the actual cause of death could not be determined with any degree of accuracy in high proportion of cases. 

**Figure 2:**
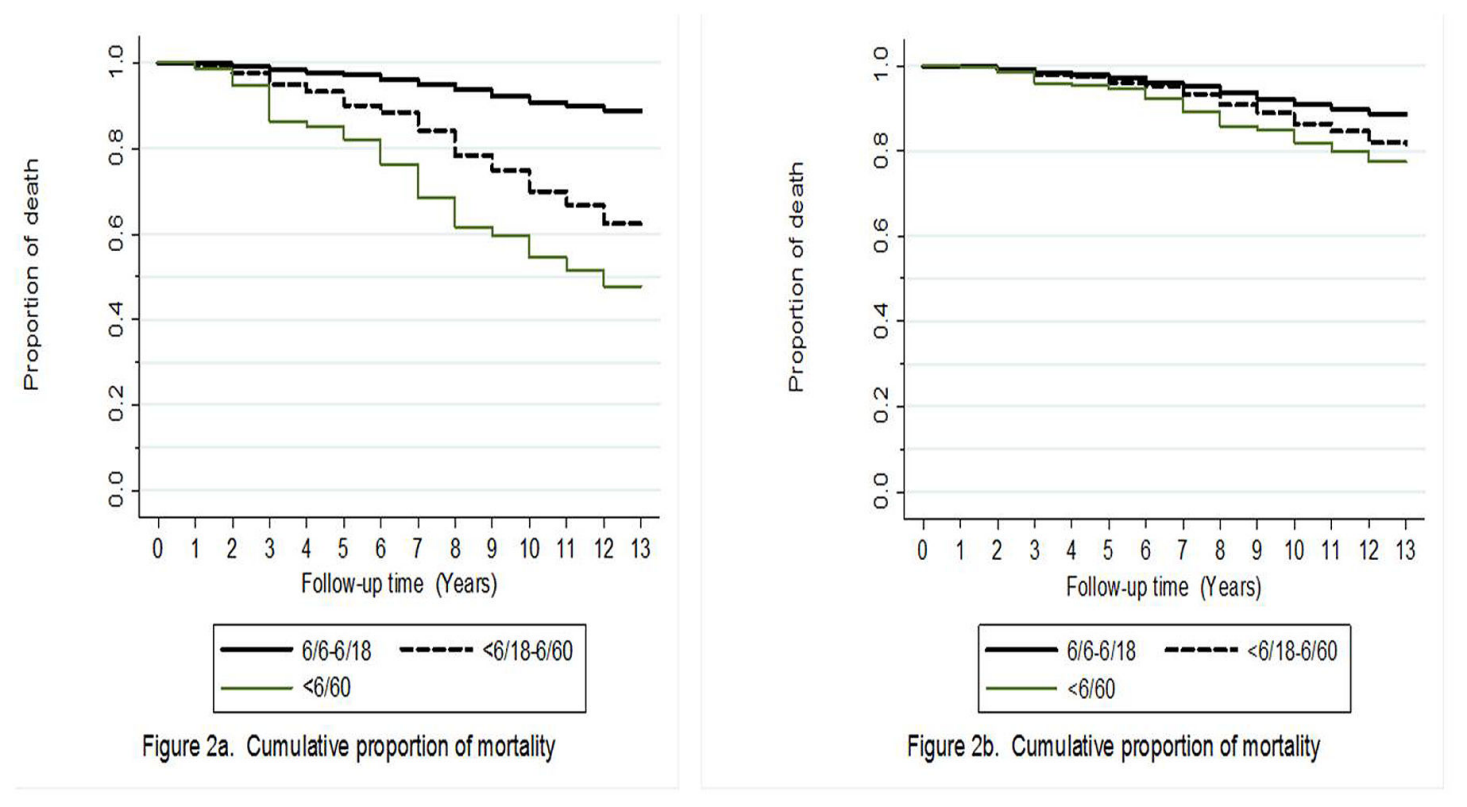
Survival curves for visual impairment and mortality: a) Unadjusted and b) Adjusted for age and gender.

**Figure 3:**
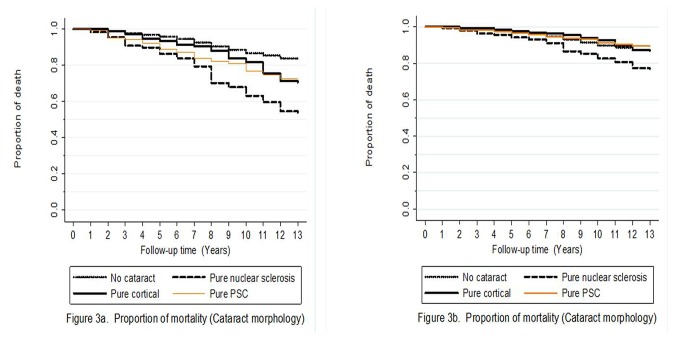
Survival curves for cataract morphology and mortality: a) Unadjusted and b) Adjusted for age and gender.

Life table graphs showing the probability of death by follow up time (unadjusted and adjusted for age and gender) and presence of VI and morphology of cataract at baseline are shown in figures 2 and 3. Participants with VI or blindness (uncontrolled and after controlling for age and gender) were at increase risk of mortality (*P*<0.001;Figure 2). Similarly, those with pure nuclear, cortical and PSC were also at increase risk of mortality than those without any cataract (*P*<0.001). 

## Discussion

In this population based study in Andhra Pradesh, India, the 10-12 year cumulative mortality in those aged 30 years and above at baseline was 19.1% (1.7%/year). This was lower than other longitudinal population based studies, such as the Beaver Dam Study (BDS) (32%; age group 43-84 years and 14 years follow-up) [6], Blue Mountain Eye Study (BMES) (28.9%; age group 49 years or older; 11 years follow-up) [3] and The Copenhagen City Eye Study (CCES) (60.9%; age group 60-80 years; 14 years follow-up) [1] (Table 6). In these studies, all participants were older at baseline, and follow up intervals varied. Analysis of APEDS2 data using comparable ages at APEDS1 to those in the other studies showed that average annual mortality rates were similar, being slightly higher in our study, For example, in BMES, which also had 11 years of follow up, mortality among those aged 43-84 years in was 28.9% compared to 36.3% in APEDS2. As the data was not age-standardized, the differences may reflect the varied demographics differences between samples as well as differences in life expectancy. 

**Table 6:** The incidence of mortality and duration of follow up duration across different studies.

**Sr. No**	**Population-based studies** (**year of publication**)	**Follow-up duration ( No. of years)**	**Age group (Years)**	**No. of participants at baseline**	**Incidence of mortality**: **N (%**)
1	Framingham Heart Study (1985)[ 16]	4.8-8	52-85	1945	312 (16)
2	The Beaver Dam Eye Study (1995)[ 5]	5	43-84	4926	467 (9.5)
3	Salisbury Eye Evaluation (1997)[ 18]	2	65-84	2520	147 (5.8)
4	Barbados Eye Study (2001)[ 14]	4	40-84	4709	306 (6.8)
5	Melbourne Visual Impairment Project (2001)[ 8]	5	40-98	3271	231 (7.1)
6	Blue Mountain Eye Study (2001)[ 11]	5	>=49	3654	604 (16.5)
7	North London Eye Study (2002)[ 17]	4	>65	1502	222 (14.7)
8	The Rotterdam Study (2003)[ 13]	7	>=55	6339	1359 (21.4)
9	Priverno Eye Study (2004)[ 15]	7	45-69	860	44 (5.1)
10	Age Related Eye Disease Study (2004)[ 2]	9	55-81	4753	534 (11)
11	The Copenhagen City Eye Study (2005)[ 1]	14	60-80	964	577 (60.9)
12	The Beaver Dam Eye Study (2006)[ 6]	14	43-84	4926	1576 (32)
13	Blue Mountain Eye Study (2007)[ 3]	11	>=49	3654	1039 (28.9)
14	Tanjong Pagar Study (2008)[ 4]	7	>=40	1232	126 (10.2)
15	Beijing Eye Study (2008)[ 12]	5	>=40	4439	143 (3.2)
16	Harbin Eye Study (2011)[ 7]	4	50-96	5057	214 (4.2)
17	APEDS2 (Present study)	11	>=30	4188	799 (19.1)

In our study we also found that VI and blindness were associated with all causes of mortality (Model 1). The association, though attenuated, held true even after including other age related eye diseases in the model (model 4). This association has been reported in previous studies (Table 7) [2-4,6-11]. The possible explanations could be that 1) visual acuity is associated with markers of frailty and frailty predicts mortality[20], 2) there may be common underlying genetic mechanisms in eye diseases and mortality [22], 3) environmental exposures may also increase the risk of eye disease and mortality, and finally, 4) there is some evidence that low visual acuity itself increases the risk of mortality through falls, accidents and depression [21,23,24]. A further explanation is that the findings may be due to residual confounding, as we were only able to adjust for a limited number of variables. 

**Table 7:** The risk factors for mortality across different studies.

**Sr. No **	**Population-based studies**	**VI / blindness**	**Nuclear**	**Cortical**	**PSC**	**Mixed**	**Any cataract **	**Cataract surgery**	**ARMD**
1	Framingham Heart Study (1985)[16]	NA	+ in diabetics	+ in diabetics	+ in diabetics	NA	NA	NA	NA
2	The Beaver Dam Eye Study (1995)[5]	-	+	-	-	NA	-	-	-
3	Salisbury Eye Evaluation (1997)[18]	NA	+	-	-	+	NA	-	NA
4	Barbados Eye Study (2001)[14]	NA	+	-	-	+	NA	NA	NA
5	Melbourne Visual Impairment Project (2001)[8]	+	-	+	-	NA	NA	-	-
6	Blue Mountain Study (2001)[11]	+	+	+	+	NA	NA	-	-
7	North London Eye Study (2002)[17]	NA	+ in non-diabetics women	+ in non-diabetics women	+ in non-diabetics women	NA	NA	NA	NA
8	The Rotterdam Study (2003)[13]	NA	-	-	-	-	-	-	-
9	Priverno Eye Study (2004)[15]	NA	+	-	-	-	NA	+	NA
10	Age Related Eye Disease Study (2004)[2]	+	+	-	-	NA	+	+	+ in advance ARMD
11	The Copenhagen City Eye Study (2005)[1]	-	NA	NA	NA	NA	-	NA	+ for women
12	The Beaver Dam Eye Study (2006)[6]	+	+	+	-	NA	+	-	-
13	Blue Mountain Study (2007)[3]	+ for <75 years	+	+	+	NA	+	-	+ for <75 years
14	Tanjong Pagar Study (2008)[4]	+	-	-	-	NA	NA	-	NA
15	Beijing Eye Study (2008)[12]	-	-	-	-	NA	NA	NA	NA
16	Harbin Eye Study (2011)[7]	+	NA	NA	NA	NA	NA	NA	NA
17	APEDS2 (Present study)	+	+	+	-	+	+	+	-

+ significant association; - Insignificant association; NA : Data not available

Similar to most studies (Table 7; including long term studies such as BDS, BMES), we found an association between mortality and nuclear cataract [2,3,5,6,11,14-18,39], cortical cataract [3,6,11,16,17] and mixed cataract [2,3,6,14,18,19] and any type of cataract with mortality [2,6,11]. The sample size was probably too small for PSC to give statistically significant findings in our study. The association between cataract and mortality has been reported across studies undertaken in different geographic locations and ethnic groups, at different times using different cataract classification systems, which suggests that biological processes are responsible for both. Changes inside the lens may reflect molecular [40], cellular and epigenetic mechanisms, including glycosulation and denaturation of proteins and oxidative stress that also impact on systemic health [22]. These mechanisms may be mediated in part by behavioural and/or environmental exposures such as cigarette smoking and ultraviolet light, which are risk factors for nuclear and cortical cataract respectively [41,42]. In India, severe dehydration has also been shown to be associated with early onset of cataract [43].

Our finding of increased mortality in individuals who underwent cataract surgery is not consistently reported in other studies (Table 7). Some studies have exhibited positive associations [2,15,44-50] while other were negative [3-6,8,11,13,18,19]. These differences could be explained by differences in study design, age at surgery, the degree of lens opacities at surgery as well as differences in follow-up rates. Some studies were hospital based rather than population based, which may bias the findings. In our study, the association of mortality with cataract surgery (Model 4) remained consistent even after adjusting for visual acuity. 

ARMD was not associated with mortality in our study after adjusting for confounders, which probably reflects the low prevalence of the disease in the population and the small number of cases (Table 7).

The large population based sample and high response rates are major strengths of our study. A limitation is that change in exposure status over time was not assessed, but this would also have reduced the magnitude of the effect. Data on the causes of mortality also depended on verbal autopsy and many relatives reported symptoms rather than diseases, which limited the data on causes of mortality. However, despite these limitations, our observations are of importance as this is the first time that such data have been reported from a rural setting in Asia where life expectancy, access to health care services and risk factors are very different from the developed nations. Our findings support the hypothesis that cataract reflect systemic ageing and that lens may be a useful biomarker of ageing [22].
